# Cell‐cycle regulation of sarcomere integrity—Role for Actn2 phosphorylation

**DOI:** 10.14814/phy2.70826

**Published:** 2026-04-09

**Authors:** S. S. Baksh, I. Anwar, X. Wang, R. E. Pratt, V. J. Dzau, C. P. Hodgkinson

**Affiliations:** ^1^ Mandel Center for Heart and Vascular Research, and the Duke Cardiovascular Research Center Duke University Medical Center Durham North Carolina USA

**Keywords:** Actn2, cell cycle, gene editing, phosphorylation, sarcomere

## Abstract

Sarcomeres are the fundamental contractile units of muscle. Despite their importance, sarcomere assembly remains poorly understood. We focused on Actn2, a protein which stabilizes the sarcomere by linking proteins to the Z‐disk. During C2C12 differentiation into myoblasts, Actn2 protein levels remained constant. This finding suggested that Actn2 incorporation into the sarcomere arose from a post‐translational mechanism. We hypothesized that the post‐translational mechanism relied on phosphorylation. Alignment of Actn2 protein sequences from animals with three‐ or four‐chambered hearts identified a conserved sequence, T^308^P^309^E^310^K^311^, that matches the consensus phosphorylation motif of the cell‐cycle kinase CDK1. In vitro kinase assays showed that CDK1 phosphorylates Actn2 at T308. In contrast, CDK1 was unable to phosphorylate Actn2 when T308 was mutated to alanine (T308A). Using CRISPR‐Cas9 gene editing, we created Actn2‐T308A and phosphomimetic Actn2‐T308D variants in C2C12 cells. C2C12 cells expressing Actn2‐T308A differentiated rapidly and formed robust sarcomeres. However, C2C12 cells expressing Actn2‐T308D failed to form organized sarcomeres. Curiously, Actn2‐T308A cells were found to have less proliferative capacity than Actn2‐T308D cells. Taken together, these results identify CDK1‐dependent phosphorylation of Actn2‐T308 as being important for sarcomere assembly. Moreover, the data also suggest a mechanism by which cell‐cycle exit promotes sarcomere assembly.

## 1 INTRODUCTION

The sarcomere is the smallest contractile unit of muscle tissue (Herzog, 2022). Structurally, sarcomeres are highly organized macromolecular complexes with actin and myosin filaments at their core. Proper sarcomere assembly is essential for muscle contraction. Disruptions in sarcomere assembly cause a range of myopathies (Keyt et al., 2022; Noureddine & Gehmlich, 2023). Beyond contraction, sarcomeres also impact muscle regeneration. Adult mammalian hearts fail to regenerate after injury partly because cardiac muscle cells cannot re‐enter the cell cycle. The failure to re‐enter the cell cycle is linked to an inability to induce sarcomere disassembly (Pettinato et al., 2022). Despite the importance of sarcomere assembly for muscle function and regeneration, mechanisms remain poorly understood.

Sarcomeres are complex, with various components, including thin and thick filaments as well as a structure known as the Z‐disk. The Z‐disk plays an important role as an anchor for thin filaments. Moreover, the Z‐disk is connected to myosin thick filaments through Titin. Myosin thick filaments align with actin thin filaments to form the contractile lattice. During contraction, myosin heads pull actin filaments toward the sarcomere center, generating force via the sliding filament mechanism. Among Z‐disk proteins, Actn2 plays several important roles. Actn2 links actin filaments to the Z‐disk, forms a critical point of contact with Titin, and provides a scaffold for signaling molecules. Together, these roles make Actn2 essential for sarcomere assembly and function (Linke, 2023; Roper, 2025; Solis & Russell, 2021).

This study aimed to determine how phosphorylation regulates sarcomere assembly. Although phosphorylation is known to influence sarcomere assembly, the mechanisms remain poorly defined. In the C2C12 model of skeletal muscle differentiation, Actn2 levels remained constant, suggesting post‐translational regulation. Sequence analysis revealed that animals with three‐ and four‐chambered hearts harbor a conserved CDK1 site at T308 in the Actn2 protein. In vitro, Actn2‐T308 was phosphorylated by CDK1. Using CRISPR‐Cas9, we generated non‐phosphorylatable (T308A) and phosphomimetic (T308D) Actn2 variants. T308A cells formed robust sarcomeres and differentiated efficiently. In contrast, T308D cells failed to assemble organized sarcomeres. T308A cells also exhibited reduced proliferation compared with T308D cells, linking Actn2 phosphorylation to the cell cycle. These results identify CDK1‐dependent phosphorylation of Actn2‐T308 as a key switch coordinating sarcomere assembly and proliferation.

## 2 MATERIALS & METHODS

### 2.1 Skeletal muscle differentiation

C2C12 cells were maintained in growth media (DMEM, 15% v/v FBS, 1% v/v penicillin–streptomycin). The cells were acquired from ATCC (catalog number: 30–2002) and routinely passaged at 70–80% confluence using 0.05% w/v trypsin. For differentiation, C2C12 cells were seeded at 75,000 cells/cm^2^ in growth media. The cells were allowed to reach confluence (day 0 of the differentiation protocol) and skeletal muscle differentiation was carried out by culturing the cells in differentiation media (DMEM, 10% v/v FBS). Media was refreshed every 48 h (Harris et al., 2024).

### 2.2 Immunoblotting

The cell layer was washed once with PBS, and proteins were lysed with 150 μL of lysis buffer (62.5 mM Tris pH 8, 1% vol/vol SDS, 1% vol/vol mammalian protease inhibitor cocktail; Sigma‐Aldrich) per well of a 6‐well plate on ice. Proteins were separated by SDS‐PAGE (Invitrogen) and transferred to nitrocellulose (Bio‐Rad). Nitrocellulose membranes were probed with a primary antibody overnight at 4 °C in an antibody buffer (50 mM Tris–HCl, 150 mM NaCl, 5% nonfat milk, 0.1% Tween‐20, pH 7.4). Membranes were subsequently washed three times with Tris‐buffered saline (TBS)‐Tween (50 mM Tris–HCl, 150 mM NaCl, 0.1% Tween‐20, pH 7.4) at room temperature and then incubated for 1.5 h with horseradish peroxidase (HRP)‐conjugated secondary antibody buffer (1:1000 dilution). After three washes with TBS‐Tween, proteins were visualized by chemiluminescence using ECL Plus (GE Healthcare). Band intensities were determined by the Image J or Syngene software. Primary and secondary antibodies were acquired from Sigma‐Aldrich and Cell Signaling Technology, respectively.

### 2.3 In vitro CDK1 kinase assay

Was carried out using the BPS Bioscience CDK1 Assay Kit (catalog no.: 79597) according to the manufacturer's instructions. Several CDK1 substrate peptides were used. The first was a CDK1 substrate peptide (HATPPKKKRK) supplied by the manufacturer of the kit (BPS Bioscience). The second peptide was a 21‐amino acid long peptide encompassing the Actn2 T308 amino acid (RTIPWLNER**T**PEKTMQAMQKK). By way of a control, the Actn2 T308 peptide was modified to convert T308 to A308 as the alanine cannot be phosphorylated. The peptides were manufactured by Biomatik.

### 2.4 HDR gene editing

C2C12 cells were modified by gene editing to convert the T308 amino acid of Actn2 into either A308 (T308A) or D308 (T308D). The approach employed was homology‐directed repair (HDR). HDR is a DNA repair mechanism that uses a homologous DNA sequence as a template to fix double‐strand breaks. In this study, double‐strand breaks were induced by transfecting the cells with a Cas9 expression plasmid (LentiCRISPR v2 Addgene Plasmid, no.: 52961). The template DNA for fixing the double‐stranded breaks contained the aforementioned T308 modifications in a single‐stranded DNA oligonucleotide (ssODN). The ssODN was transfected into C2C12 cells alongside the Cas9 expression plasmid. Transfection was carried out with Lipofectamine LTX with Plus reagent according to the manufacturer's (Thermo Fisher) instructions.

The ssODNs were designed such that the PAM (NGG) sequence immediately preceded target sequence and, to achieve high HDR efficiencies, flanking sequences homologous to the target region were >40 bases on each side.

*T308A*:

5′TTGGGTGGCTTGTGCTTCCGGCGGTAATCCCGGAAGTCCTCCAGCTTCTTCTGCATGGCTTGCATGGTCTTCTCGGGAGCCCGGTTCTCCAGCCAGGGGATCGTGCGACGAATCCATTCC3′.

*T308D*:

5′TTGGGTGGCTTGTGCTTCCGGCGGTAATCCCGGAAGTCCTCCAGCTTCTTCTGCATGGCTTGCATGGTCTTCTCGGGATCCCGGTTCTCCAGCCAGGGGATCGTGCGACGAATCCATTCC3′.

Successful insertion of the HDR construct was determined by a custom TaqMan SNP Genotyping Assay (Thermo Fisher). Sequences were not supplied.

### 2.5 Immunostaining

Cells were fixed with 4% PFA and incubated overnight at 4 °C with Actn2 antibody (1:50 in antibody buffer (1xPBS, 0.1% Tween‐20, 5% BSA)). Following the overnight incubation, cells were washed three times in antibody buffer (5 min per wash, room temperature) and subsequently incubated for a further hour with a 1:100 dilution of an Alexa‐Fluor 594 antibody (cat no.: A21201; Thermo Fisher) in antibody buffer at room temperature. In the last 30 min of the incubation, DAPI was added to a final concentration of 1 μg/ml to stain nuclei. Following washing in PBS to remove unbound complexes, immunofluorescence was measured using a Zeiss Axiovert 200 inverted microscope. Areas occupied by Actn2 and DAPI staining were determined by Image J.

### 2.6 Images

Images were prepared with the CorelDraw and Zeiss software (Axiovision Rel4.8 and Zen Blue) 3.

### 2.7 Statistics

All statistical analysis was performed with GraphPad. Two‐tailed *t*‐tests (two groups) and one‐way ANOVAs (> two groups) were used as appropriate. For ANOVA, Bonferroni post hoc tests were used to determine significance between groups.

## 3 RESULTS

**FIGURE 1:**
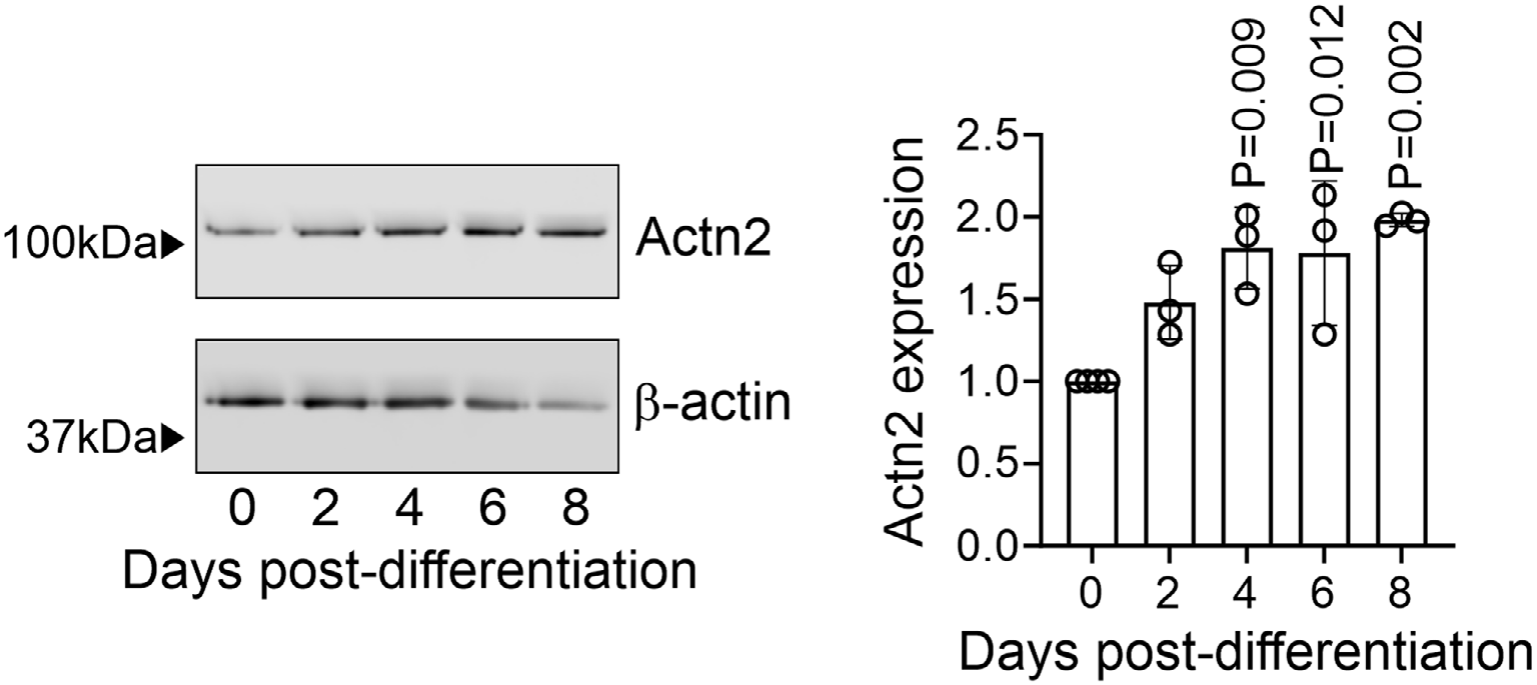
Actn2 expression during muscle differentiation. C2C12 cells were differentiated to myotubes according to standard protocols. At the indicated times, protein extracts were analyzed for Actn2 expression. For comparison, β‐Actin levels are shown. The graph shows the relative expression of Actn2 for three independent experiments. Statistics: One‐way ANOVA with Bonferroni post hoc tests. *p < 0.05, **p <0.01.

To investigate the mechanisms of sarcomere assembly, we focused on Actn2 (Gomez et al., 2021; Hodgkinson et al., 2018; Hsueh et al., 2023). Actn2 is found in both skeletal and cardiac muscle where it functions to anchor myofibrillar actin thin filaments and Titin to Z‐disks. Actn2 is also believed to play a role in docking signaling molecules to Z‐disks (Lek et al., 2010; Murphy & Young, 2015). Actn2 protein levels were found to be relatively constant during skeletal muscle differentiation (Figure 1), suggesting that incorporation of Actn2 into the Z‐disk is dependent on a post‐translational mechanism.

**FIGURE 2:**
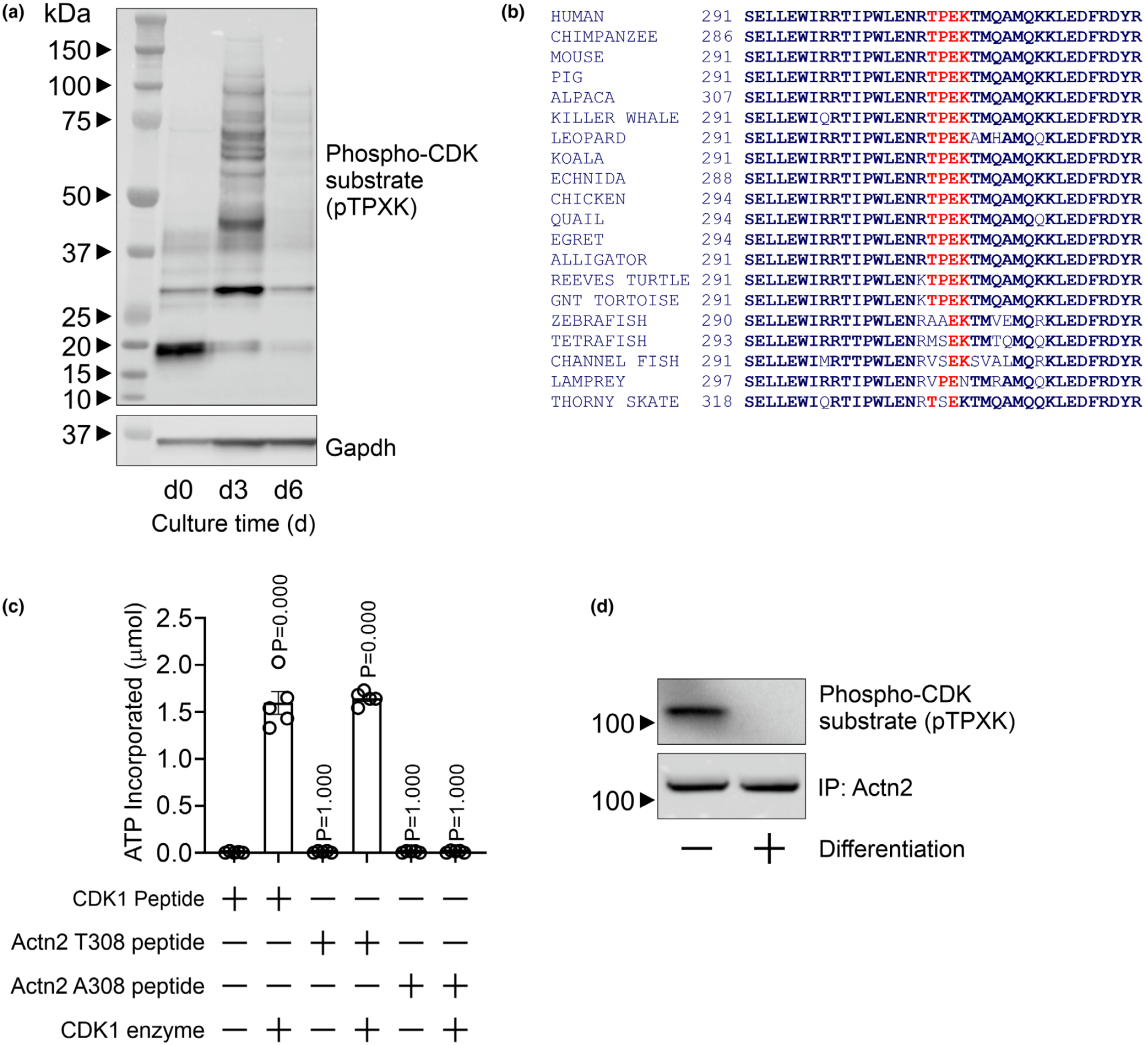
Actn2 is phosphorylated by CDK1 at amino acid T308. (a) iPSCs were differentiated under standard conditions to generate cardiomyocytes. Protein extracts were isolated at the indicated times in the differentiation process and analyzed with an antibody detecting CDK1 phosphorylated proteins. GAPDH was used as a loading control. (b) CDK1 phosphorylates substrates at a S/TPx(x)R/K consensus motif. Only one sequence in Actn2 matches this consensus motif: TPEK. TPEK is conserved in all species with a three‐ and four‐chambered heart. (c) CDK1 kinase assay: CDK1 enzyme was incubated with an Actn2 peptide (21‐mer) containing the TPEK consensus motif. A positive control and negative control peptide were used. The negative control was a scrambled Actn2 peptide, whereby TPEK was converted to APEK. Peptide phosphorylation was determined by measuring the incorporation of a fluorescently tagged ATP. Standard curves were used to convert absorbance to molar amount. *N* = 5. (d) Actn2 was immunoprecipitated from iPSC‐derived cardiac progenitors or iPSC‐derived cardiomyocytes. Immunoprecipitates were probed with an antibody which detects CDK1 phosphorylated proteins. Representative immunoblots from three independent experiments.

To identify the post‐translational mechanism, we focused on phosphorylation. Cell‐cycle withdrawal occurs early during muscle cell differentiation as shown by an analysis of cell‐cycle kinase activity. We observed that CDK1 robustly phosphorylates target proteins early in the differentiation of iPSCs to cardiomyocytes before activity disappears in later stages of differentiation (Figure 2a). We asked whether Actn2 contained CDK1 phosphorylation site(s). The consensus CDK1 phosphorylation site has the sequence S/TPx(x)R/K, where S/T is the amino acid directly phosphorylated. Human Actn2 contains one potential CDK1 phosphorylation motif, T^308^P^309^E^310^K^311^. Interestingly, the TPEK motif is present in all animals with a three‐ or four‐chambered heart (Figure 2b). To determine whether the Actn2 T308 amino acid was phosphorylated by CDK1, in vitro kinase assays were employed. Several peptides were used. The first was a control CDK1 substrate peptide (HATPPKKKRK) supplied by the manufacturer of the kit (BPS Bioscience). The second peptide was a 21 amino acid long peptide encompassing the Actn2 T308 amino acid (RTIPWLNER**T**PEKTMQAMQKK). By way of a control, the Actn2 T308 peptide was modified to convert T308 to A308 as the alanine cannot be phosphorylated. The synthetic 21 amino acid peptide containing the Actn2 TPEK motif was robustly phosphorylated by CDK1 (Figure 2c). Phosphorylation of the Actn2 T308 peptide was robust and equivalent to the positive control (Figure 2c). Importantly, CDK1 phosphorylation was lost when T308 was substituted with alanine (Figure 2c), indicating that phosphorylation only occurred on T308 and not anywhere else in the peptide. To provide further evidence, iPSCs were analyzed for Actn2 T308 phosphorylation during their differentiation to cardiomyocytes. IPSC‐derived cardiac progenitors (proliferative cells devoid of sarcomeres) were found to contain phosphorylated Actn2 T308. In contrast, in iPSC‐derived cardiomyocytes (non‐proliferative cells with mature sarcomeres), Actn2 T308 phosphorylation was not detected (Figure 2d).

**FIGURE 3:**
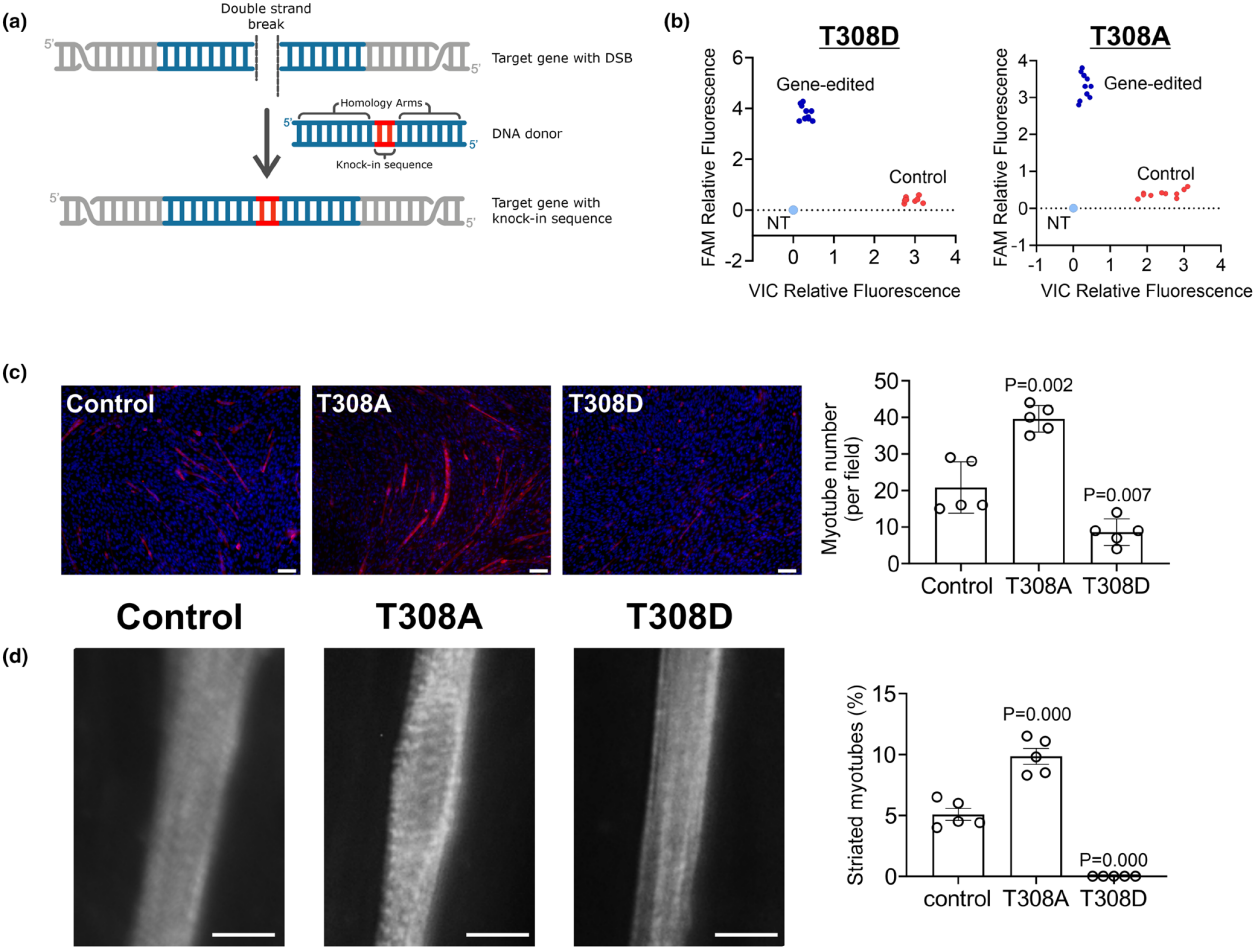
Preventing Actn2 T308 phosphorylation by gene editing promotes C2C12 myotube differentiation and sarcomere formation. (a) Overview of the CRISPR‐Cas9 homology‐directed repair (HDR) approach. (b–d) C2C12 cells were transfected with Cas9 and the gRNAs needed to convert the Actn2 T308 residue to A308 (prevents phosphorylation) or D308 (mimics phosphorylation). After 7 days, cells were analyzed for gene editing or seeded for differentiation. Analysis of gene‐editing efficiency (panel b) was determined by qPCR with primers designed for the introduced SNP. Differentiation was assessed 6 days after seeding. In the first instance (panel c), myotube number was calculated by staining for Actn2. Results of five independent experiments are shown. For each experiment, four images per well were taken and the average myotube number reported. A further analysis of differentiation was carried out by calculating the percentage of myotubes displaying striated sarcomeres (panel d). *N* = 5. Statistics: One‐way ANOVA with Bonferroni post hoc testing, **p < 0.01, ***p < 0.001.

To provide further evidence of the role of Actn2 T308 phosphorylation in sarcomere formation, we turned to CRISPR‐Cas9 homology‐directed repair (HDR) gene editing (Figure 3a). HDR gene editing was used to convert T308 to alanine (T308A) or aspartic acid (T308D). Converting T308 to A308 (T308A) prevents CDK1 phosphorylation, while converting T308 to D308 (T308D) mimics CDK1 phosphorylation. In vitro kinase assays demonstrated (Figure 2c) that Actn2 T308A cannot be phosphorylated by CDK1. HDR gene editing of C9C12 cells was robust (Figure 3b). When compared to control cells expressing the wild‐type Actn2 T308, cells expressing the Actn2 T308A variant were found to differentiate to myotubes at a more rapid pace (Figure 3c). In contrast, cells expressing Actn2 T308D, mimicking the phosphorylated state, displayed a weak capacity for differentiation (Figure 3c). These differences were further borne out when investigating sarcomeres. Sarcomere formation was robust in cells expressing Actn2 T308A and absent in cells expressing Actn2 T308D (Figure 3d).

**FIGURE 4:**
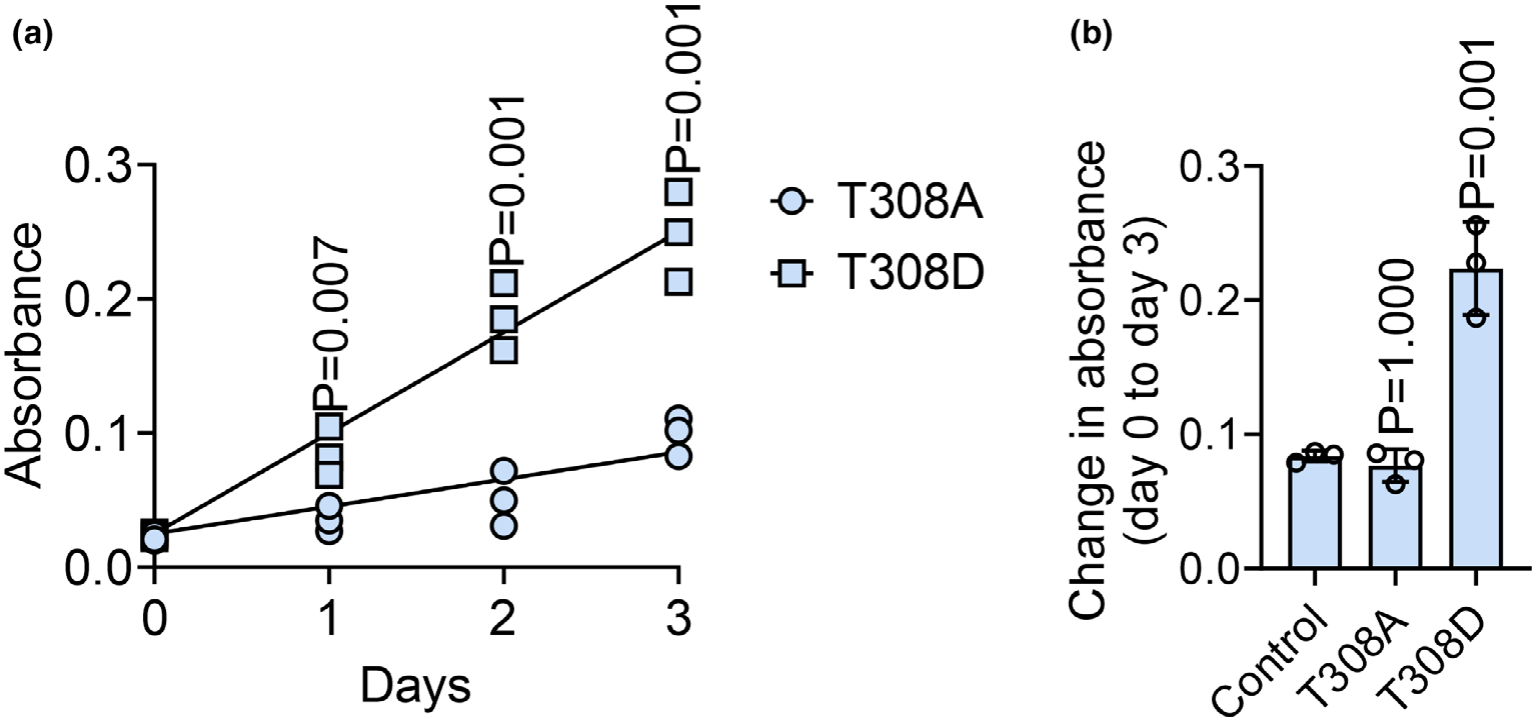
Actn2 T308 phosphorylation correlates with increased proliferative capacity. C2C12 cells were transfected with the Actn2 T308A or Actn2 T308D expression plasmids. Cell number was determined by MTS assay at the indicated times post‐transfection. *N* = 3. ***p < 0.001.

Finally, we wanted to determine the impact of Actn2 phosphorylation on proliferation. We observed that C2C12 cells expressing the T308A variant were less proliferative than those expressing the T308D variant (Figure 4). This observation implies that Actn2 phosphorylation provides a feedback loop to regulate cell proliferation.

## 4 DISCUSSION

Considering their role in muscle contraction, there has been much interest in identifying the mechanisms of sarcomere assembly (Luis & Schnorrer, 2021; Martin & Kirk, 2020; Nayak & Amrute‐Nayak, 2020). Deciphering mechanisms has proven to be a difficult task as the sarcomere is highly complex, where more than 20 proteins have to be precisely located within the structure (Gregorio et al., 1999; Martin & Kirk, 2020; Prill & Dawson, 2020; Rui et al., 2010; Sparrow & Schock, 2009; Thievessen et al., 2022). Increasing evidence suggests that phosphorylation plays a central regulatory role. During the early stages of myofibrillogenesis, phosphorylation of Z‐disk‐associated proteins, such as Actn2 by kinases, including PKC and CaMKII appears to be necessary for Z‐disk formation and function. Additional kinases, such as MURF1/2, DMPK, and PKD, also localize to developing sarcomeres, where they are thought to coordinate Z‐disk maturation through phosphorylation‐dependent signaling. In parallel, phosphorylation also appears to be needed for thick‐ and thin‐filament formation. Thick‐filament assembly and alignment is promoted by MLCK phosphorylation of Myosin regulatory light chain. Phosphorylation of troponin and tropomyosin by PKA and PKC modifies thin‐filament behavior to fine‐tune early contractile behavior. As sarcomeres mature, phosphorylation of titin by PKA, PKG, PKC, and CaMKII adjusts elasticity and binding properties, contributing to tension homeostasis and mechanical stability (Curtis et al., 2023; Kampourakis & Irving, 2015; Muller et al., 2014; Reimann et al., 2017; Seguchi et al., 2007; Xiao et al., 2022; Yamasaki et al., 2002).

Phosphoproteomic studies of differentiating muscle cells suggest that many kinases involved in sarcomere assembly remain to be identified. In that light, our data identify an important role for CDK1. In C2C12 cells modified to express the phosphomimetic Actn2 variant T308D, sarcomeres were unable to form. Positioning this finding within the existing phosphorylation schema as outlined above, CDK1‐mediated Actn2 phosphorylation would appear to be in the early assembly and Z‐disk formation stage, but with a unique inhibitory role. While PKC or CaMKII phosphorylation of Actn2 aids Z‐disk formation, CDK1 phosphorylation appears to block Z‐disk formation entirely. This suggests a distinction between assembly‐permissive phosphorylation (by muscle kinases) and assembly‐preventive phosphorylation (by cell‐cycle kinases).

Our finding that Actn2 is phosphorylated by CDK1 provides a potential link between sarcomere assembly and the cell cycle. CDK1 activity is high during cell proliferation and declines as cells exit the cycle and initiate differentiation. The observation that CDK1‐dependent phosphorylation of Actn2 prevents sarcomere formation suggests that this modification may act as a mechanism to delay sarcomere formation until cells become post‐mitotic. In this context, phosphorylation could serve to maintain Actn2 in a state that is incompatible with stable Z‐disk formation, thereby ensuring that cytoskeletal remodeling does not occur concurrently with cell division. In other words, CDK1‐mediated Actn2 phosphorylation would represent a gatekeeping mechanism, coupling the transition from proliferative to differentiated states. This has important implications for regenerative medicine as the inability of adult mammalian hearts to replenish the muscle population after injury is believed to be the reason why people do not recover from heart attacks (Bersell et al., 2009; D'Uva et al., 2015; Fan et al., 2015; Mahmoud et al., 2013; Nguyen et al., 2020; Porrello et al., 2011).

Support for our work can be found in the study of Fan et al. (2015). Here, the authors showed that mitosis in neonatal cardiomyocyte induced sarcomere disassembly and Z‐disk collapse. Notably, mitosis induced Actn2 phosphorylation. While the authors were unable to pinpoint the phosphorylation site or the kinase responsible, it is notable that a CDK1 inhibitor led to sarcomere reassembly. Unfortunately, the authors did not determine whether the CDK1 inhibitor led to Actn2 dephosphorylation. However, Actn2 phosphorylation peaked in G2/M where CDK1 is most active.

**FIGURE 5:**
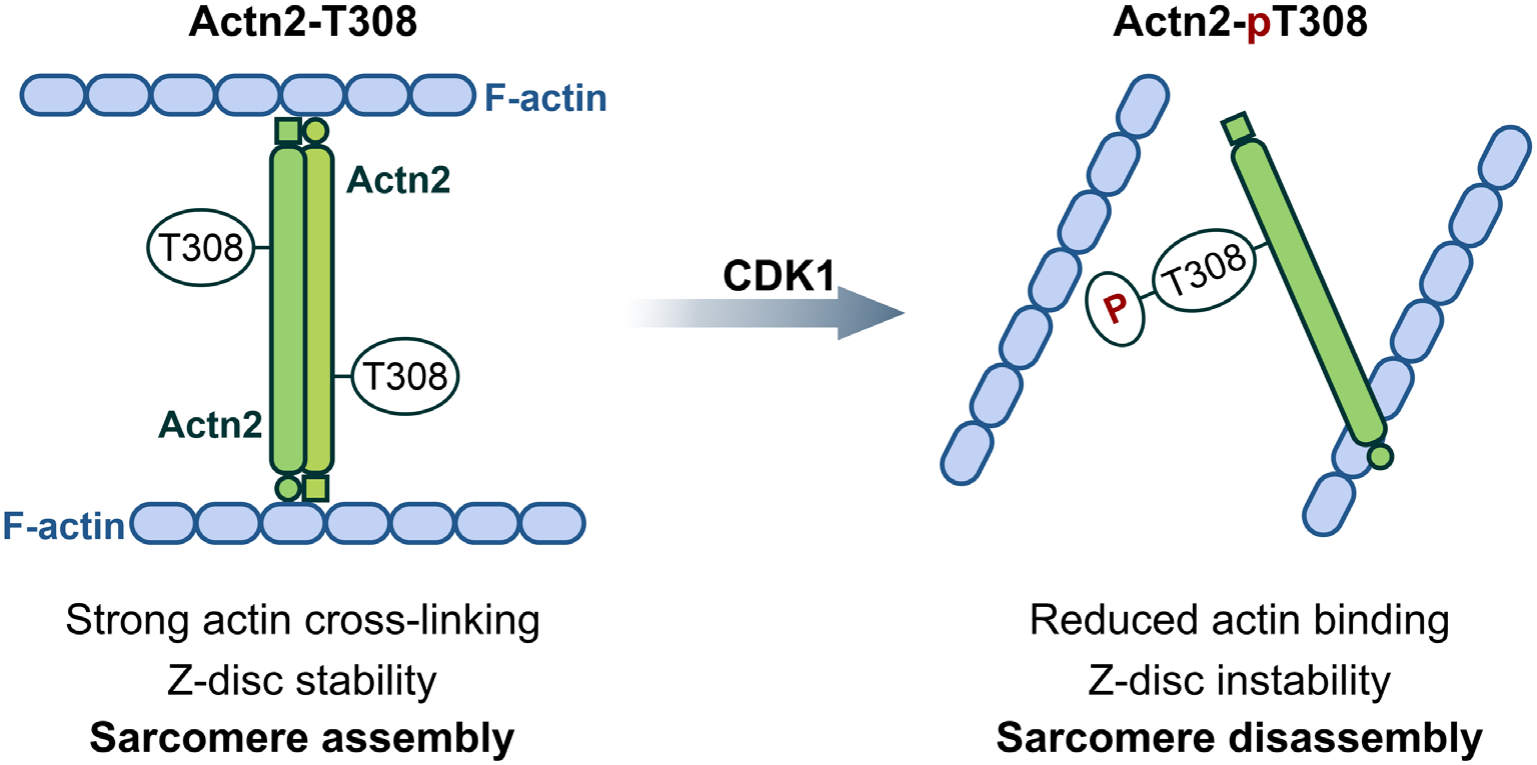
Actn2 T308 phosphorylation and sarcomere assembly. The data provided in this study suggests a mechanism whereby Actn2 T308 phosphorylation regulates sarcomere assembly. In the presence of the cell‐cycle kinase CDK1, Actn2 T308 is phosphorylated and sarcomeres become unstable. When cells exit the cell‐cycle and CDK1 activity declines, Actn2 T308 is dephosphorylated. In the dephosphorylated state, Actn2 promotes sarcomere formation.

In summary, our study reframes phosphorylation not merely as a fine‐tuning mechanism but as a binary developmental switch. During proliferation, CDK1 activity is high, Actn2 is phosphorylated, and sarcomere assembly is repressed. When cells exit the cell cycle to differentiate, CDK1 is inactivated, Actn2 is dephosphorylated, and sarcomere assembly is permitted. Long hypothesized but not previously shown, the study directly connects sarcomere formation to the cell cycle (Figure 5).

## AUTHOR CONTRIBUTIONS

**S. S. Baksh:** Data curation; investigation; methodology. **I. Anwar:** Data curation; formal analysis; investigation. **X. Wang:** Data curation; investigation; methodology. **R. E. Pratt:** Data curation; investigation; methodology. **V. J. Dzau:** Data curation; funding acquisition; investigation; methodology. **C. P. Hodgkinson:** Conceptualization; data curation; investigation; methodology; project administration.

## FUNDING INFORMATION

This work was supported by the NHLBI of the National Institutes of Health (Grant No. R01‐HL178582 to IA, XW, REP, VJD, and CPH). The content is solely the responsibility of the authors and does not necessarily represent the official views of the National Institutes of Health.

## CONFLICT OF INTEREST STATEMENT

The authors report no conflicts of interest.

## ETHICS STATEMENT

All research was sanctioned by the Division of Laboratory Animals (DLAR) at Duke University, as well as the Duke Institutional Animal Care and Use Committee (IACUC).

## Data Availability

All of the data are presented in this manuscript.
